# Soft Gel-Based Transformative Structured Engineering Design

**DOI:** 10.1021/accountsmr.4c00348

**Published:** 2025-11-21

**Authors:** Kaixin Jiang, Xue Chen, Chunyan Dai, Ben Bin Xu

**Affiliations:** † Key Laboratory of Tropical Medicinal Resource Chemistry of Ministry of Education, Key Laboratory of Tropical Medicinal Plant Chemistry of Hainan Province, The International Joint Research Center for Clean and Efficient Utilization of Hydrocarbon Resources in the South China Sea of Hainan Province, Engineering Research Center of Tropical Marine Functional Polymer Materials of Hainan Province, Key Laboratory of Functional Organic Polymers of Haikou, 12389 Hainan Normal University, Haikou 571158, P.R. China; ‡ School of Engineering, Physics and Mathematics, 5995 Northumbria University, Newcastle Upon Tyne NE 18ST, United Kingdom

## Abstract

Soft gels, a category of soft materials, consist of polymer networks with small molecules, such as water or other solvents. They possess mechanical flexibility and softness along with tunable physical and chemical functionalities. These gels are capable of responding to external stimuli, such as temperature, pH, light, and electric and magnetic fields, making them highly suitable for applications in drug delivery, tissue engineering, sensors, and soft robotics. As many advantages as soft gels have, there are many more mechanisms to be understood to bridge clear structure–function relationships. There is also a continuous need to facilitate these new functionalities into the device or product technologies. In this Account, we aim to provide an overview of recent progress in functional soft gels with a focus on structural design and innovative fabrication techniques. We start with exploring how structural design can impart diverse functionalities to soft gels. This is followed by a discussion of mechanics with an emphasis on elastic instabilities that are deliberately introduced and controlled to achieve shape morphing. The multilength scale instabilities will be linked with local to global surface deformation and/or macroscopic deformation of gel objects. We then examine how chemical modifications—especially cross-linking and network formation—contribute to the architecture and functionality of soft gels. These chemical modifications have been harnessed to enrich the designability of the gel to enable extra function or provide dedicated controllability. Manufacturing techniques also play a vital role in establishing structural varieties that enable programmable responses to external stimuli for specific applications. We offer a quick scan on the frontier technologies on fabricating soft gel-based devices with an alignment to the advanced manufacturing trend with novelty structural design. Finally, the applications of functional soft gels were selectively scoped in areas such as sensing, energy and sustainable materials, and biomedical devices. They are well-suited for both diagnostic and therapeutic functions. All the above applications will be enabled by the novel structural design with realization of unique structure–property relationships. Designed structures can be programmed to exhibit specific mechanical behaviors, which, in turn, enable responsive and functional soft gels. Importantly, when a stimulus activates the designated trigger points, the engineered structure responds in the manner that we designed. This interplay within the gel ultimately manifests as a controllable response, highlighting how transformative structural engineering serves as the foundation for achieving multifunctionality. We conclude by highlighting the current challenges and future directions in the development of high-performance functional soft gels through structure-based design.

## Graphical abstract

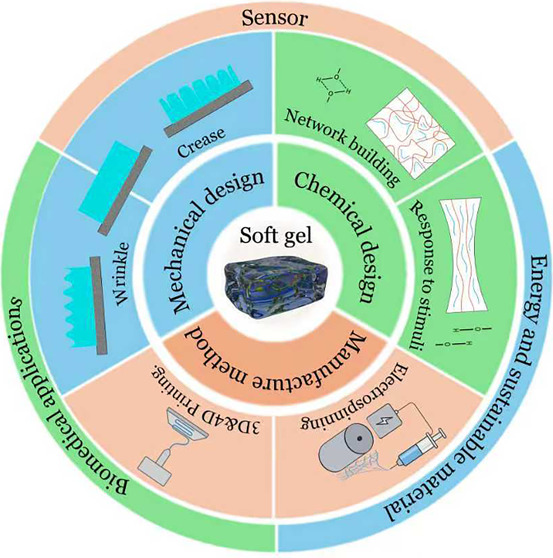

## 1 Introduction

Soft gels are a unique class of materials that combine the softness and deformability with added functionalities such as high electrical conductivity, responsiveness to external stimuli, or enhanced mechanical strength.^1–4^ These gels (Figure 1a,b) consist of polymeric networks and liquid molecules, typically water or organic solvents, allowing them to maintain a soft, flexible structure while serving as a platform for advanced applications. To achieve multifunctions, these gels are often integrated with nanomaterials or functional chemicals that impart specific properties (Figure 1c).^5–7^ The special groups and chemical bonds in the soft gels respond to environmental stimuli and exhibit unique responsive mechanical behavior like wrinkles and creases through structure design (Figure 1d). Together, these components allow functional soft gels to serve as versatile platforms in emerging technologies ranging from wearable sensors and soft robotics to biomedical devices and energy storage systems.

**Figure 1:**
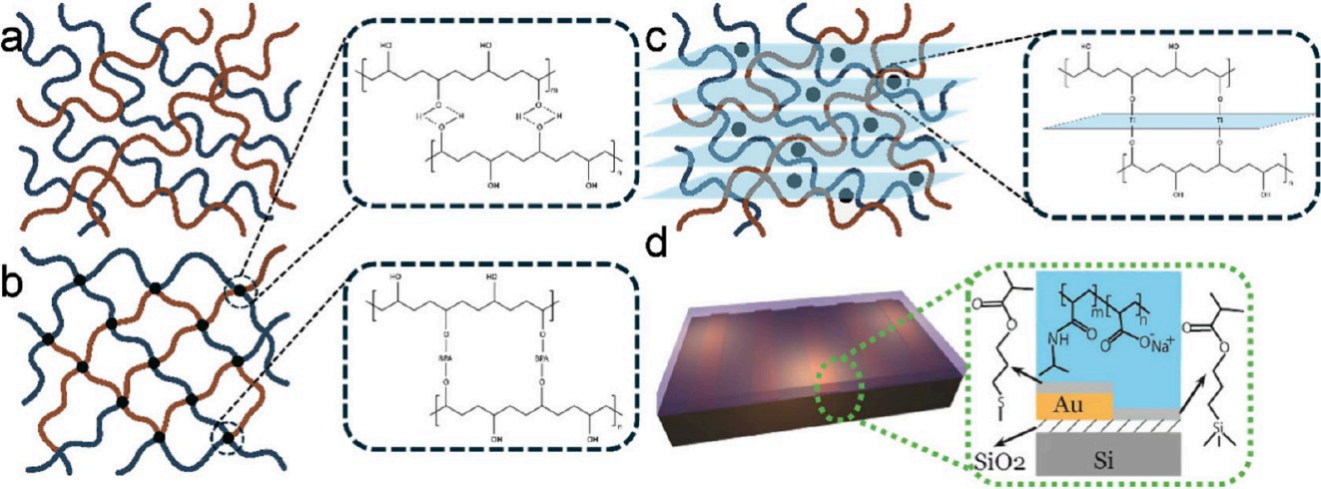
(a) Interpenetrating polymer network structure of polymers in gels. (b) Cross-link structure formed by hydrogen bond and covalent bond. (c) 2D materials such as MXene and graphene incorporated in gels. (d) Example of the anionic poly­(*N*-isopropylacrylamide-*co*-sodium acrylate) (PNIPAM) copolymer hydrogel. Reproduced with permission from ref 4. Copyright 2013 Wiley-VCH.

In this Account, we review the unique design and structural engineering of functional gels for advanced applications. We start with the overview of the chemical/physical properties in gel. Then, we discuss different design directions and realization strategies to achieve functional gels, including elastic instability, network building, and manufacture. Next, we scope the recent development of functional gel applications in sensing strategies, energy and sustainable materials, and medical applications. At the end, we give a conclusion and outlook of the functional structure design of gel materials.

## 2 The Physical and Chemical Properties of Gels

Soft gels exhibit nonlinear mechanical behavior,^8^ like strain stiffening or softening, and can be engineered to display self-healing and shape-memory effects. These behaviors are often the result of dynamic interactions within the polymer network, such as reversible hydrogen bonding, ionic cross-links, or supramolecular assembly. The result is a material that not only deforms easily but also recovers its shape, heals damage, or adapts to changing conditions—properties that are increasingly critical in soft multifunctional systems.

Functional soft gels exhibit a unique combination of extreme softness and multifunctionality, positioning them at the forefront of adaptive material systems. One of their defining physical characteristics is the exceptional elasticity with an elastic modulus ranging from a few kilopascals to several hundred kilopascals, which enables them to conform to complex surfaces and undergo large deformations, similar to natural tissues. However, this mechanical compliance also makes gels susceptible to elastic instabilities, which arise when the system relieves internal stresses by transitioning from smooth to patterned or nonuniform states.^9^ Wrinkling often occurs in bilayer systems where a stiff skin overlays a soft substrate, leading to periodic surface undulations under compressive strain. Buckling and folding may occur in slender gel structures or thin films when axial stresses exceed a critical threshold, while creasing typically emerges as sharp surface indentations when the material undergoes large localized strains. Traditionally considered mechanical failures, these phenomena are now being harnessed in soft gels for advanced functionalities, such as dynamically tunable optics, reconfigurable adhesion, smart surface texturing, and mechanical signal amplification. The spontaneous pattern formation resulting from elastic instability provides a powerful mechanism for generating complex, reversible deformations without the need for intricate fabrication processes. Furthermore, gels frequently exhibit nonlinear mechanical behaviors, such as strain stiffening or softening, broadening their capacity for sophisticated mechanical responses.

To achieve controllable and reversible shape morphing, structural design is crucial. We can encode deformation pathways into gels by introducing anisotropic architectures, patterning gradients of cross-linking density, or creating multilayer structures with differing mechanical responses. These designs enable the gel to transform into preprogrammed shapes or exhibit targeted motion when stimulated by environmental changes, such as temperature, humidity, or mechanical loading. Importantly, the structural approach controls how elastic instabilities develop and localize, guiding the formation of wrinkles, folds, or other mechanical instabilities in a predictable and useful way. This method allows soft gels to function as shape-shifting materials in soft robotics, wearable devices, and biomedical interfaces, where precisely directed mechanical responses are required.

Chemical modification is central not only to expanding gel functionality but also to programming how the network architecture dictates mechanical responsiveness. By introducing functional moieties or orthogonal chemistries, chemical reactions embed responsive triggers in response to pH, temperature, ionic strength, redox conditions, or light^10^ into the network, enabling hierarchical or multinetwork structures that change their properties under defined conditions. These modifications directly influence stiffness, elasticity, and viscoelastic dissipation, allowing the gel’s mechanical profile to be tuned with molecular precision. Chemical heterogeneity, introduced deliberately through functionalization, determines whether gels resist or yield under small versus large deformation or whether they dissipate or store energy under fast versus slow loading. Cross-linking chemistry is particularly decisive: permanent covalent bonds generate durable frameworks, while dynamic interactions—such as metal–ligand coordination, ionic pairing, or host–guest recognition—function as built-in molecular switches that trigger adaptability. These dynamic cross-links act as responsive nodes, enabling gels to self-heal, relax stress, or reconfigure their architecture in response to stimuli.

The combination of mechanical programmability through structural design and molecular-level control through chemical modification enables soft gels to exhibit complex, multifunctional behaviors. While elastic instability provides a means of achieving dynamic shape transformation and pattern generation via physical design, chemical modification introduces direct responsiveness to environmental stimuli, allowing gels to adapt their properties, heal, or actively interact with biological and artificial systems. Together, these strategies establish soft gels as versatile platforms for applications in soft robotics, bioelectronics, and adaptive biomedical devices.

## 3 Design and Development of Novel Functional Gel

### 3.1 Advanced Mechanics in the Functional Gel

Elastic instability occurs locally or sectionally when the material undergoes large deformations, often leading to buckling or collapse.^11^ When an elastomer bilayer is compressed, surface wrinkles on the stiff skin layer form to alleviate in-plane compression. As compression increases, these wrinkles undergo a sequence of bifurcations (also known as Ruga evolution), potentially manifesting in period-doubling, quadrupling, and eventually deep folds or creases. The wrinkling and postwrinkling behaviors are influenced by intrinsic material properties such as modulus disparity and Poisson’s ratio as well as structural factors like layer thickness and prestrain conditions.

Soft gels undergo mechanical instabilities when subjected to compressive, tensile, or swelling-induced stresses, often transitioning from a smooth to a patterned surface to release accumulated internal stress. As the internal elastic energy builds, through solvent-induced swelling, mechanical compression, or interfacial adhesion, the gel may relieve this stress by forming surface instabilities. A commonly studied configuration involves a cross-linked polymer gel layer adhered to a rigid substrate. This is typically prepared by polymerizing the gel directly on the substrate, followed by immersion in a solvent. Initially stress-free, the gel begins to swell as the solvent penetrates, generating osmotic pressure due to the mixing of solvent molecules with polymer chains and counterions. This pressure is counterbalanced by the elastic restoring force of the polymer network. Because of the mechanical constraint provided by the substrate, a surface-attached gel that is much thinner than its lateral dimensions can expand only in the direction normal to the surface. The result of this uniaxial expansion is that the gel experiences an equibiaxial compressive stress. This stress can be described using a neo-Hookean strain energy density function:

$$W=\frac{\mu }{2}\left(tr\left(F^{T}F\right)-3\right)$$ 1

where *W* is the strain energy density, μ is the shear modulus of the gel, and *F* is the deformation gradient tensor. For incompressible gels, det*F* = 1. This equation captures how elastic energy builds up under deformation When the in-plane compressive strain reaches a critical value, the system becomes unstable and surface patterns emerge. In the case of a thin stiff film on a soft substrate, the critical strain for wrinkling can be approximated by

$$\begin{array}{c} \varepsilon_{cr}^{wrinkle}=\frac{2}{3}{\left(\frac{E_{f}}{E_{s}}\right)}^{2/3} \end{array}$$ 2

where *E*_f_ and *E*_s_ are the Young’s moduli of the film and substrate, respectively. The corresponding wrinkle wavelength is given by

$$\begin{array}{c} \lambda =2\pi h_{f}{\left(\frac{E_{f}}{3E_{s}}\right)}^{1/3} \end{array}$$ 3

with *h*_f_ being the thickness of the film. As the compression continues to increase, the system may evolve beyond smooth wrinkling into sharp folds or creases. In homogeneous gels, creasing typically appears when the compressive strain exceeds approximately:

$$\begin{array}{c} \varepsilon_{cr}^{crease}\approx 0.35 \end{array}$$ 4

This transition is highly nonlinear and often involves self-contact, marking a distinct form of elastic instability. For surface-attached films, the resulting compressive stresses can be large—often comparable to or greater than the gel’s elastic modulus. When this happens and delamination is suppressed, the surface folds to relieve stress (Figure 2a,b),^12^ forming a characteristic pattern where fold spacing is nearly equal to the gel thickness.

**Figure 2:**
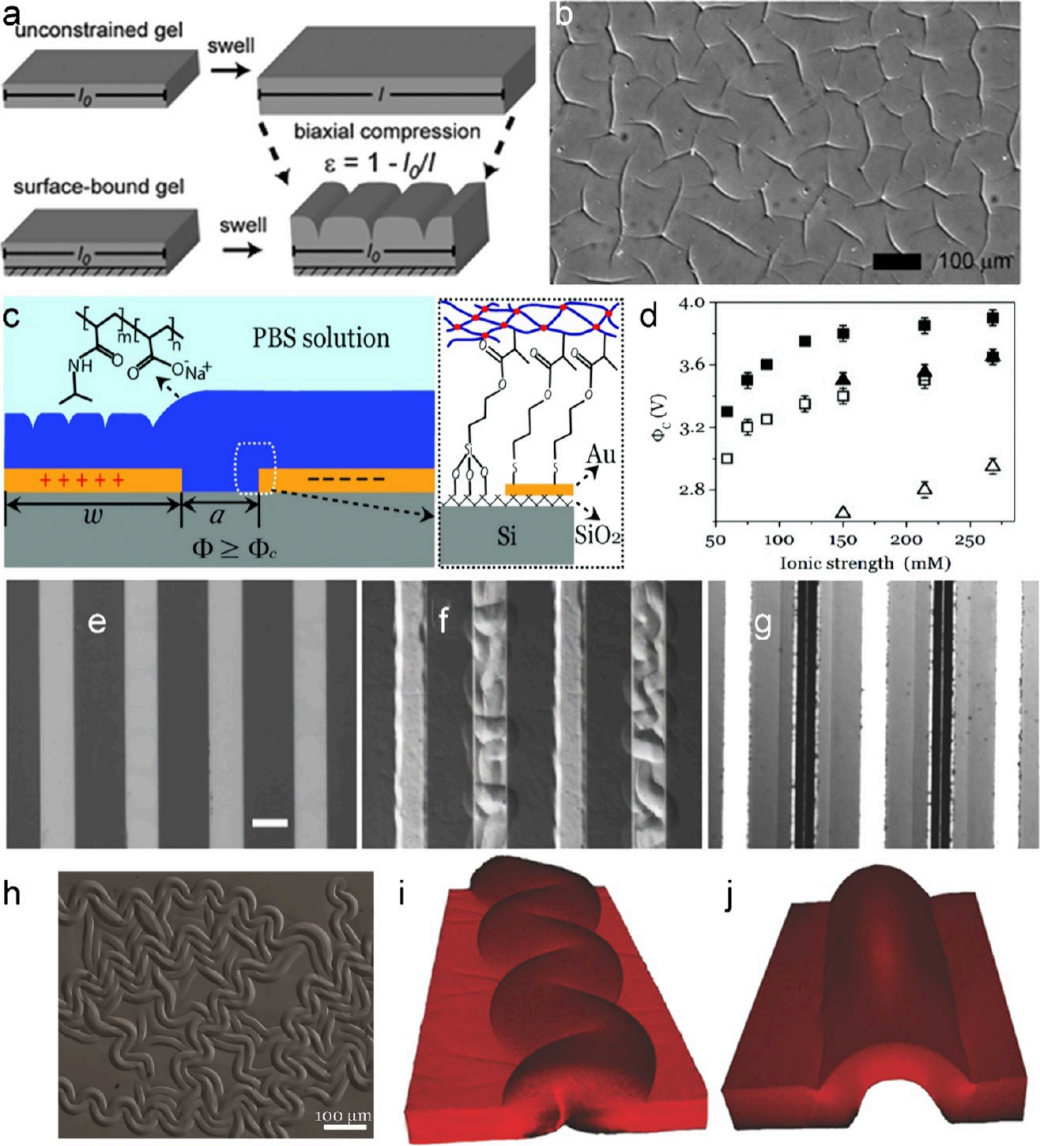
(a) Biaxial compressive stress develops when a surface-attached gel swells in one direction, and this stress can be partially alleviated through the formation of localized creases on the gel surface. (b) An optical micrograph of the crease structures formed upon swelling of a surface-attached gel. Reproduced with permission from ref 12. Copyright 2008 The Royal Society of Chemistry. (c) An illustration of the device geometry, adhesion-promoting self-assembled monolayers, and chemical composition of the anionic PNIPAM copolymer hydrogel. (d) The critical voltage to form creases as a function of ionic strength for *h* = 13 μm (open symbols) and *h* = 25 μm (filled symbols) at *T* = 30 °C (triangles) and 40 °C (squares). Reproduced with permission from ref 4. Copyright 2013 Wiley-VCH. (e–j) Experimental characterization of two-stage delamination buckling. (e) While the surface is initially flat, increasing the applied potential results in (f) stage I—delamination and buckling, followed by (g) stage II—formation of a straight-sided “Euler column”. (h) Extensive delamination of a hydrogel film swelled to *H*/*h* = 1.9 on a silicon wafer without silane treatment. The scale bar is 50 μm. LSCM 3D reconstructed images show (i) stage I delamination exhibiting a regular “telephone cord” morphology and (j) stage II Euler column. Reproduced with permission from ref 13. Copyright 2016 Wiley-VCH.

We developed an approach to achieve rapidly switchable and completely reversible changes in the surface of the PNIPAM hydrogels with patterns that can be precisely controlled through the electrode geometry (Figure 2c).^4^ Above a critical voltage, surface creases formed selectively above the anode, while the cathode region remained flat. The creases form selectively above the anode, suggesting that localized electrochemical reactions at the anode lead to differential swelling and mechanical instability. This phenomenon is attributed to the electro-osmotic flow and ion redistribution near the anode, causing localized expansion and subsequent surface buckling. The critical voltage for crease formation increased with temperature and ionic strength and slightly with gel thickness (Figure 2d), reflecting changes in the gel’s swelling behavior and electrical properties. However, this phenomenon clearly indicates that electrically driven creasing is not simply explained by local changes in swelling, since (at least far from the electrode edge) a decrease in volumetric swelling will lower the compressive stress at the surface.

We further studied electrically triggered delamination and buckling of anionic PNIPAM copolymer gel surfaces supported on silicon substrates.^13^ A electrochemical delamination and the subsequent buckling process was observed in a soft gel film on the top interdigitated electrodes (Figure 2e–j). As the applied DC voltage increased, initial changes occurred over the cathode: the gel slightly swelled due to electrochemical modulation, followed by Stage I delamination at around −4 V. This stage involves the gel locally detaching and buckling out-of-plane, forming blisters that rapidly spread and persist even after the voltage is turned off. Imaging reveals shallow gaps and self-contact between the gel and substrate, along with distinct surface patterns such as irregular buckling or periodic “telephone-cord” shapes. The onset of Stage I delamination is linked to reductive desorption of thiol monolayers bonding the gel to the cathode, as confirmed by cyclic voltammetry. However, delamination depends not only on redox processes but also on compressive stresses within the gel. At a higher voltage (Stage II), the gel undergoes further transformation into straight-sided blisters (“Euler columns”), which are wider and deeper and display pronounced optical contrast, indicating large gaps and gas accumulation. This stage is driven by hydrogen gas bubble formation from water electrolysis, which rapidly inflates the delaminated regions. Both stages are sensitive to the gel’s swelling ratio (*H*/*h*), showing that mechanical stress and elastic energy play key roles alongside electrochemical effects. The morphology of blisters is shaped by mechanical constraints—notably, the need to conserve volume during deformation—and by water transport into the gel and the delaminated region. This behavior parallels observations in other soft systems, where volume constraints and swelling govern postdelamination shape evolution.

Elastic instability can be deliberately induced and controlled to achieve a structural surface, self-assembled structures, and tunable mechanical properties.^13–15^ In multiscale systems, elastic instabilities involve complex interactions across different scales, particularly in composite materials.^16^ These instabilities arise when a material or structure transitions between stable configurations under external stimuli such as mechanical loads, temperature fluctuations, or environmental factors. The behavior of elastic instabilities is highly dependent on scale, as interactions at the micro-, meso-, and macrolevels influence the global deformation response. Microstructural features, including pores, fibers, and interfacial layers, play a key role in determining the overall mechanical behavior. Localized stress concentrations at the micro- and mesoscales often lead to wrinkling, creasing, or folding, which then propagate into large-scale buckling or long-wave deformation patterns at the macroscale.^17^

Elastic instabilities offer transformative potential across multiple fields. In soft robotics and actuators, controlled instability enables programmable deformations, allowing for adaptive movement and shape morphing.^18–20^ In biomedical engineering, elastic instabilities have been exploited to develop wearable devices, tunable drug delivery systems, and biointegrated sensors.^21–24^ The ability to harness instabilities in a controlled manner is particularly relevant in microfluidics, where surface instabilities can be used to manipulate fluid flow, self-cleaning surfaces, and microscale patterning.^14,25,26^ Elastic instabilities play a crucial role in biological systems. Many natural structures, including plant leaves, marine organisms, and human skin, exhibit wrinkling and buckling patterns that enhance functionality. Understanding these natural instability mechanisms has inspired biomimetic designs such as adaptive camouflage materials and responsive surfaces for medical applications.

By integration of insights from multiscale modeling, experimental techniques, and theoretical frameworks, researchers can harness elastic instabilities to design advanced functional materials and adaptive structures. Whether in mechanical metamaterials, biomedical applications, or soft robotics, the ability to control and exploit instabilities presents significant opportunities for next-generation technologies. Future research will focus on refining computational models, improving fabrication techniques, and expanding real-world applications, further advancing the field of elastic instability engineering.

### 3.2 Chemical Modification Drives Functional Gels

Chemical modification allows for the design of cross-linking strategies that dictate the gel’s architecture. Gel networks are constructed through a combination of permanent covalent cross-links and dynamic or reversible bonds. Covalent bonds form a stable and stiff framework that maintains the overall structural integrity of the gel. In contrast, dynamic cross-links enable the gel to react to environmental stimuli such as pH, temperature, redox conditions, or light. Together, these two types of interactions create a functional and responsive gel network, where the covalent backbone ensures mechanical stability, while the dynamic components provide adaptable behavior and stimulus-triggered responses.

Our group developed a highly conductive and mechanically robust electrically conductive hydrogel by *in situ* growing Ni/Co-Bimetallic zeolitic imidazolate frameworks within the poly­(vinyl alcohol)/sodium alginate dual network.^27^ Glutaraldehyde plays a role as the cross-link part, while the dual network of Poly­(vinyl alcohol) (PVA)/sodium alginate (SA) was built through the physical cross-linking of PVA and SA via van der Waals forces and hydrogen bonding. Another example is the synthesis of multifunctional polysaccharide-based bioadhesive hydrogels by incorporating carboxymethyl chitosan (CMCS) and acrylic acid *N*-hydroxysuccinimide ester (AA-NHS) into the allyl cellulose (AC) network using a light-activated strategy (Figure 3a).^28^ As illustrated in Figure 3b, the formation of a dual cross-linked network involves both chemical and physical interactions: AC and AA-NHS undergo homopolymerization or copolymerization through free radical polymerization, while AA-NHS also reacts with CMCS via NHS ester–amine coupling. Beyond the chemical cross-links, a physically cross-linked network forms through hydrogen bonding, driven by residual hydroxyl groups on the AC chains and the abundant hydroxyl, carboxyl, and amino groups present on CMCS. A stiff covalent network provides mechanical strength, while functional groups such as hydroxyl, amino, and succinimide impart strong adhesiveness, enabling the gel to combine durability with effective substrate interactions. Chen et al. proposed a supramolecular network structured polymer electrolyte to achieve good mechanical properties and high ionic conductivity.^29^ The poly­(ethylene glycol) methyl ether methacrylate, 2-(3-(6-methyl-4-oxo-1,4-dihydropyrimidin-2-yl)­ureido)­ethyl methacrylate (UPyMA), tripropylene glycol diacrylate, and 1-allyl-3-methylimidazolium bis­(trifluoromethanesulfonyl)­imide formed the skeleton of polymer electrolyte (Figure 3c). The quadruple hydrogen bonding networks constructed by the interaction of UPyMA dimers give the electrolyte a good mechanical performance.

**Figure 3:**
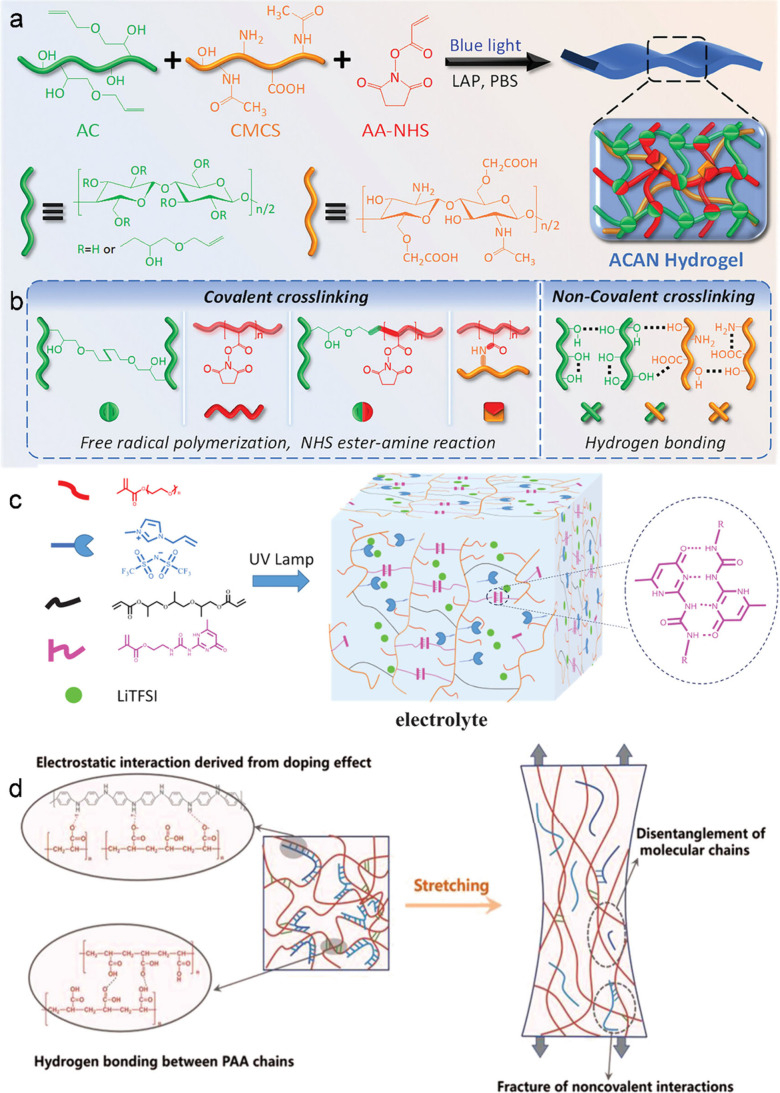
(a, b) Illustration of the hydrogel preparation and dual cross-linked network structure. Reproduced with permission from ref 28. Copyright 2024 Wiley-VCH. (c) Schematic illustration of the monomers and one-step photosynthesis of PUTP GPEs. Reproduced with permission from ref 29. Copyright 2022 Wiley-VCH. (d) Schematic illustration of the straining mechanism of the PAA/PANI hydrogel. Reproduced with permission from ref 30. Copyright 2022 Wiley-VCH.

Dynamic cross-links can be designed as a reversible part of the network, break to dissipate the applied energy, and protect the polymer chains. We proposed a novel “doping-then-gelling” strategy to fabricate an ultrastretchable supramolecular poly­(acrylic acid) (PAA)/ polyaniline (PANI) hydrogel featuring an entangled polymer network.^30^ The resulting hydrogels incorporate high-density electrostatic interactions as dynamic, reversible bonds, enabling exceptional mechanical properties, including ultrastretchability (up to 2830%), high tensile strength (120 kPa), and rapid self-healing capabilities. To better illustrate the internal network behavior under strain, a schematic is provided in Figure 3d. In this model, the dynamic noncovalent interactions between PAA and PANI chains serve as reversible “sacrificial bonds” that readily dissociate under stress, facilitating efficient energy dissipation during deformation. Simultaneously, the entangled polymer network undergoes partial disentanglement, which contributes further to energy dissipation and allows stress redistribution throughout the material. This synergistic mechanism prevents catastrophic chain scission and imparts the hydrogel with high toughness and robust self-recovery characteristics.

### 3.3 Manufacture of Functional Gels

The manufacture of soft gels has expanded beyond traditional casting to include a variety of advanced techniques that offer precise control over the structure, functionality, and responsiveness. Parameters significantly contributing to the microstructure of the gels regulate polymer chain arrangement, network density, and water content.

Electrospinning is a fast and easy to scale-up technique to produce functional materials based on polymeric fibers with diameters ranging from 2 nm to several micrometers. Under the applied electric field, the polymer solution formed a Taylor cone, and a fine jet was ejected, stretching and thinning into nano- to microscale fibers. As the jet traveled to the collector, rapid solvent evaporation led to solidification, depositing the fibers into a nonwoven mat on a grounded flat plate or rotating drum. Due to its nano/microdiameters (typically 100 nm to 5 μm), random fiber deposition results in an interconnected porous network and void spaces between fibers, creating channels for mass transport. Electrospun fibers have a high surface-to-volume ratio and tunable porosity and can be produced from natural or synthetic polymers.^31^ This structure closely mimics the extracellular matrix (ECM) and is advantageous for applications requiring rapid fluid absorption, molecular diffusion, and cell infiltration.

Yu et al. reported the unique design and fabrication of a task-specific scaffold composed of electrospun nanofibers of polymerized ionic liquid poly­(diallyldimethylammonium) bis­(trifluoromethanesulfonyl)­imide, embedded within a cross-linked poly­(2,2,2-trifluoroethyl methacrylate) matrix, to achieve a large-area polymerized ionic-liquid-nanofiber membrane.^32^ Wang et al. developed electrospun scaffolds with distinct isotropic and mechanical properties, which approximate the hierarchical fibrous layers of meniscus ECM. Electrospinning forms scaffolds with random, aligned, or yarn-like fibrous structures that mimic the hierarchical architecture of the meniscus ECM. Among these, the yarn-like configuration was particularly effective, as it supported substantial meniscus cell infiltration, which in turn enhanced scaffold mechanical properties.^33^ With the combination of other methods, the structure of soft gels can be controlled in more detail. We developed a nanofiber-based Janus membrane by electrospinning in a single-step process with high transparency (∼80–90%) and high tensile strength up to 13 MPa to promote wound healing (Figure 4a).^34^ These achievements are the result of a tailor-made design of a square pattern topological structure with a high light flux, achieved through the utilization of conductive mesh on the collector to manipulate the electrical field during fiber deposition (Figure 4b). The controlled fiber alignment and porous architecture contribute to the gel’s mechanical robustness and high breathability, enabling it to serve as an advanced wound dressing material that supports rapid wound healing.

**Figure 4:**
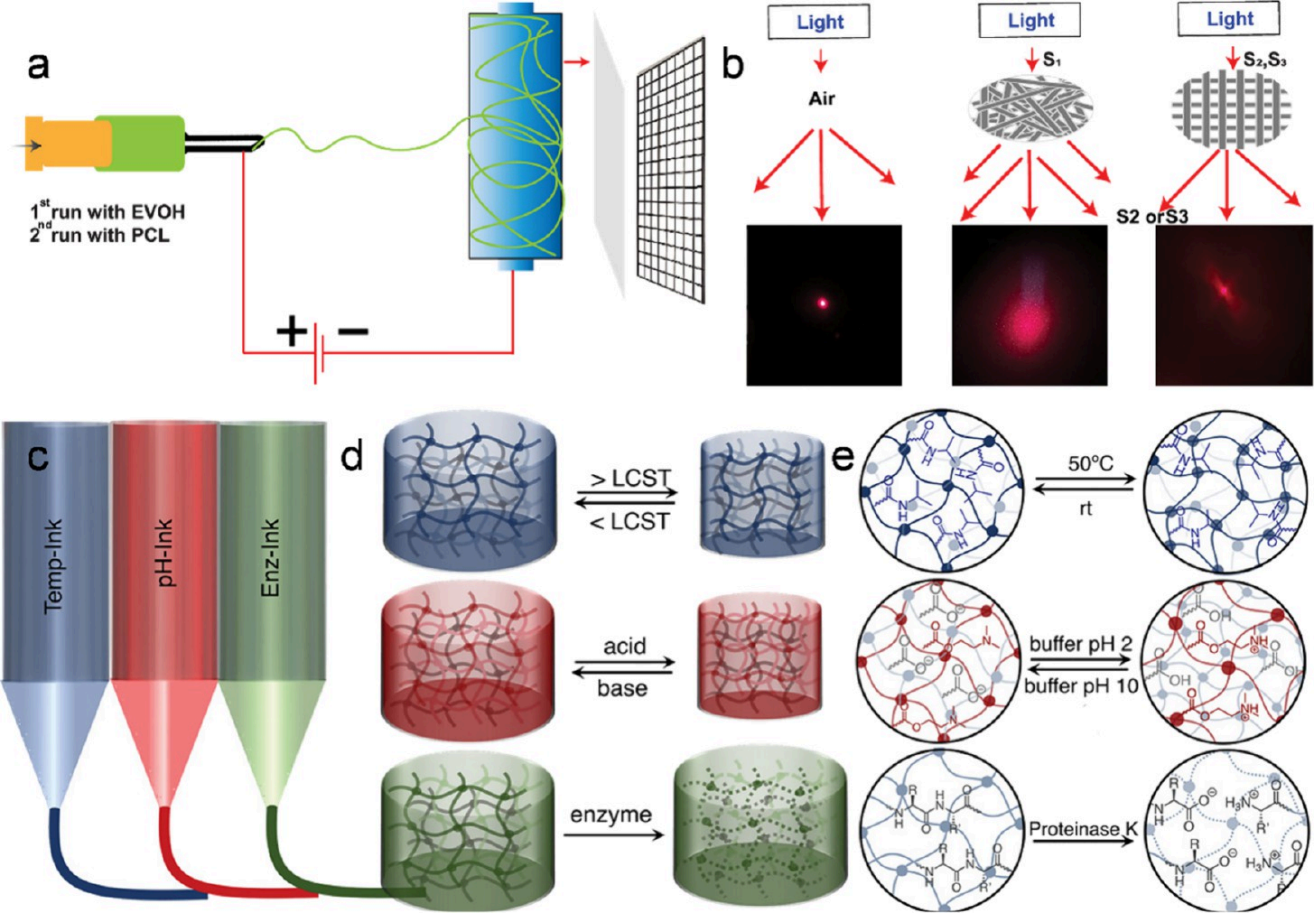
(a) Schematic illustration of the fabrication of the Janus membrane by using the electrospinning technique. (b) Photo showing the scattered laser light spot of the air, disordered fibrous membrane, and square mesh structures (S2 and S3), respectively. Reproduced from ref 34. Copyright 2024 American Chemical Society. (c) Schematic of direct ink write (DIW) 3D printing with Temp-Ink, pH-Ink, and Enz-Ink using methacrylated bovine serum albumin (MA–BSA) as a building block. (d) After photopolymerization, the Temp-Ink hydrogel exhibited reversible thermoresponsive behavior, contracting upon heating to 50 °C and expanding when cooled to room temperature. The pH-Ink hydrogel responded to pH changes by reversibly contracting in acidic environments and expanding under basic conditions. Enz-Ink hydrogel degraded in the presence of enzyme. (e) Chemical composition of hydrogels: P­(NIPAAm) for temperature response, amine groups of P­(DMAEMA) and carboxylic acid groups of MA–BSA for pH response, and amide bond of MA–BSA network for enzymatic degradation. Reproduced with permission from ref 35. Copyright 2021 Wiley-VCH.

4D printing, an advancement of 3D printing, refers to the fabrication of structures capable of changing their shape, properties, or functionality over time in response to external stimuli such as temperature, light, moisture, pH, or magnetic fields. Narupai et al. reported a 3D-printed protein-based hydrogel system that undergoes programmed shape transformations triggered by temperature, pH, and enzymatic degradation (Figure 4c–e).^35^ In this work, methacrylated bovine serum albumin (MA-BSA) served as the foundational building block to form stable Pickering emulsions in the presence of *N*-isopropylacrylamide (NIPAAm) (Temp-Ink) and 2-dimethylaminoethyl methacrylate (DMAEMA) (pH-Ink), yielding temperature- and pH-responsive hydrogels, respectively. Additionally, a third ink (Enz-Ink) was formulated using MA-BSA and poly­(ethylene oxide)-*b*-poly­(propylene oxide)-*b*-poly­(ethylene oxide) triblock copolymer (F127), functioning as a sacrificial component to enable enzymatically degradable hydrogels. Using DIW 3D printing, complex architectures were fabricated that demonstrated reversible shape changes under temperature and pH stimuli and irreversible transformation upon enzymatic degradation in a spatially programmed manner (Figure 4d,e).

## 4 Applications

### 4.1 Sensing Strategies

Hydrogels, with their swelling and deswelling behavior in response to environmental changes (such as temperature, pH, and humidity), hold huge promises for a variety of sensing applications. In particular, gel hybrids incorporating nanomaterials enhance mechanical stability, conductivity, and sensitivity, paving the way for multifunctional sensor platforms.

Hydrogels play a critical role in developing bioinspired sensors for detecting human physiological signals. Stress/strain-sensitive biosensors represent a growing field, particularly in wearable health monitoring. We described a structural gel composite (SGC) sensor that combines conductive hydrogel/MXene with a Lipogel coating for underwater sensing. The hydrophobic lipogel layer prevents swelling and ensures mechanical durability over 90 days of operation and 2000 testing cycles, maintaining a consistent gauge factor (GF) of 14.5. This innovation allows for stable, long-term mechanoreceptor performance in aquatic environments (Figure 5a,b), where the hybrid hydrogel serves as a responsive finger/elbow bending detector.^36^

**Figure 5:**
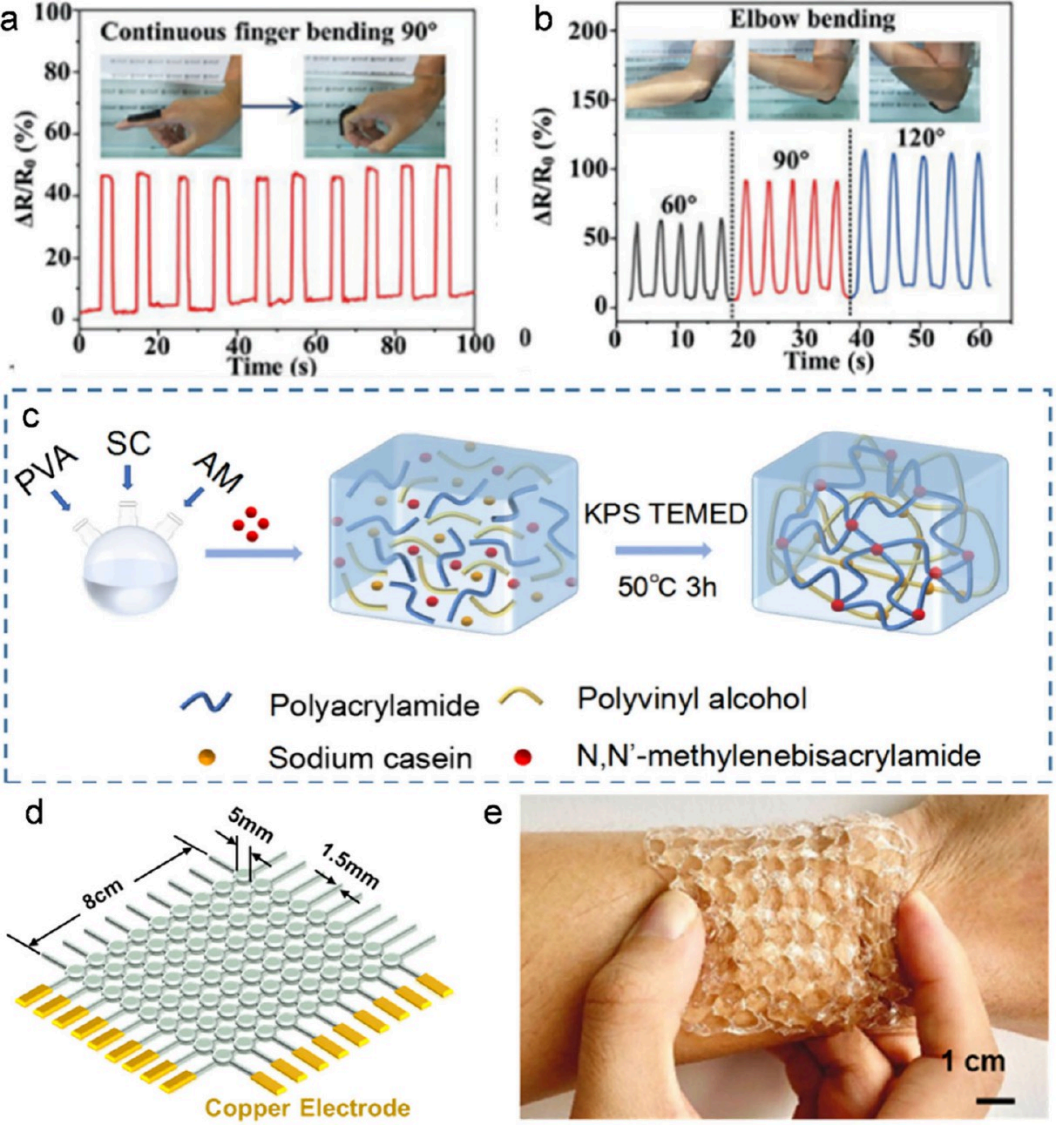
(a) SGC-based underwater strain sensors for finger bending detection and (b) elbow bending detection with various degrees. Reproduced with permission from ref 36. Copyright 2022 Wiley-VCH. (c) Illustration of the process of preparing a double-cross-linked network of hydrogels. Reproduced with permission from ref 37. Copyright 2024 Spinger Nature. (d) Three-dimensional (3D)-printed AHS1.5 hydrogel sensor array with 9 × 9 pixels (8 × 8 cm^2^). (e) Pictures of the sensor array on the wrist and under wrinkling to show its conformality with the skin. Reproduced from ref 3. Copyright 2021 American Chemical Society.

We introduced sodium casein into a dual network hydrogel consisting of PVA and polyacrylamide (PAM) to prepare an ionic hydrogel sensor (Figure 5c),^37^ which generates stable and accurate electrical signals from different locations of the human body. We also synthesized an alginate network physically cross-linked by calcium ions and semi-interpenetrating copolymers consisting of zwitterionic [2-(methacryloyloxy) ethyl] dimethyl-(3-sulfopropyl) ammonium hydroxide and 2-hydroxyethyl methacrylate.^3^ With 3D printing, sensor arrays with customizable structures were fabricated to detect force or temperature signals in 1-D and 2-D. The superior gelation, unique shear-induced gel–sol transition, and rapid self-healing capability fulfill a direct extrusion-based 3D printability to the precursor. A 9 × 9 pixel array (8 × 8 cm^2^) (Figure 5d) was rapidly fabricated in 5 min with an excellent conformability to skin (Figure 5e). The outstanding resistance change ratios from these pixel arrays are recorded and reproduced in a computer.

The integration of flexible electronics, hydrogels, and nanomaterial-based composites into next-generation sensors enables high-performance biomedical, environmental, and robotic applications. Theoretical models provide insights into mechanical, electrical, and optical transduction mechanisms, guiding the design of highly sensitive, durable, and adaptable sensors. With continuous advancements in materials science and computational intelligence, these novel sensors hold the potential to revolutionize wearable healthcare, soft robotics, environmental monitoring, and space exploration.

### 4.2 Energy and Sustainable Materials

Functional gels often based on polymers or hybrid organic–inorganic networks can conduct ions or electrons, respond to external stimuli, and incorporate active materials, making them ideal for next-generation energy systems and sustainable technologies.^38^ In energy storage devices, such as batteries and supercapacitors, functional gels often serve as electrolytes or separators. Unlike traditional liquid electrolytes, gel electrolytes reduce leakage, improve safety, and offer better mechanical stability. They can maintain high ionic conductivity while being flexible and conformable, which is particularly beneficial in flexible or wearable electronics.^39^ Chen et al. developed a transparent, flexible, moldable, and antifreezing hydrogel electrolyte with excellent mechanical strength by converting cotton into cellulose and cross-linking it with tetraethyl orthosilicate.^40^ Our group developed a new multifunctional zwitterionic monomer (ACHPES) and novel zwitterionic hydrogels.^41^ The inherent zwitterionic structure of polymer endowed it with excellent stimulus-responsive capabilities, and the introduction of long hydrophobic carbon chains between these two motifs can repel water molecules, allowing the polymer chains to fill the gaps between water molecules, hindering the formation of ice crystals, and significantly lowering the freezing point (Figure 6a). Yang et al. fabricated a new type of zwitterionic polymer hydrogel (polySH) electrolytes (Figure 6b).^42^ They added LiCl to impede the hydrogen bonds between water molecules and form a Li^+^(H_2_O)_*n*_ hydration structure with H_2_O, which can hop and migrate through the channel of the zwitterionic group, thereby improving the conductivity at low temperature (Figure 6c). In fuel cells and solar cells, functional gels can enhance the transport of protons or electrons, stabilize catalysts, and improve device efficiency.^42–44^

**Figure 6:**
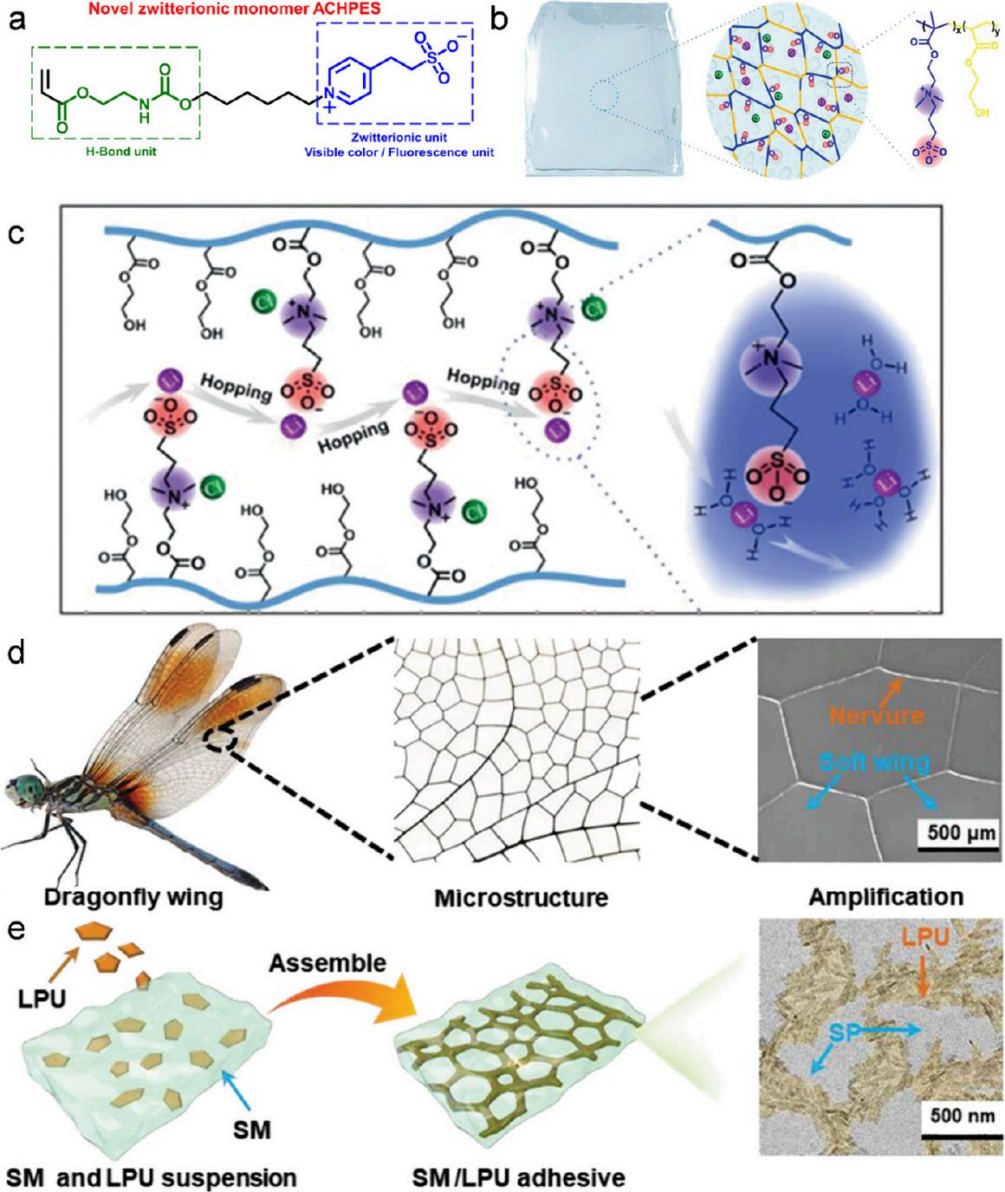
(a) Zwitterionic monomer ACHPES. Reproduced with permission from ref 41. Copyright 2025 Wiley-VCH. (b) Schematic diagram of the antifreezing polySH hydrogel and its network structure. (c) Proposed Li^+^ migration mechanism in polySH electrolyte, where hydrated Li^+^ hops through SO^3–^ sites. Reproduced with permission from ref 42. Copyright 2021 Wiley-VCH. (d) Dragonfly wing pattern with a microscopic morphology. (e) Nanostructure of the composite consisting of the LPU skeleton and SP matrix. Reproduced with permission from ref 45. Copyright 2024 Wiley-VCH.

From the sustainability perspective, these gels can be made from biodegradable or biobased materials, reducing environmental impact. They can also be designed to be recyclable, self-healing, or responsive to environmental stimuli, thus extending the life of materials and reducing waste. We designed and developed a strong and tough adhesive with excellent reprocessability by creating a dynamic network consisting of a lignin polyurea (LPU) framework with soybean protein (SP).^45^ Inspired by the microscopic structure of dragonfly wings and the strengthening and toughening mechanism, we deliberately deigned the energy dissipation path in the adhesive (Figure 6d,e). Additionally, they play a role in environmental remediation, such as capturing pollutants or aiding in water purification through selective absorption or chemical transformation.^46,47^

### 4.3 Biomedical Application

Functional soft gels have impactful applications in the medical field due to their soft, hydrated nature, biocompatibility, and tunable physical and chemical properties. Their ability to mimic the mechanical behavior of natural tissues, respond to physiological conditions, and incorporate therapeutic agents makes them ideal for both diagnostic and therapeutic use. One major application is in drug delivery systems, where soft gels can encapsulate and release drugs in a controlled, sustained, or stimuli-responsive manner. Chen et al. reported nanofibers, Tannic acid-Cellulose nanofibers (TA-CNF) and Ag nanoparticles combined with vaccarin-loaded electrospun nanofibers, to form a bilayer nanocomposite hydrogel. It enables sustained drug release and mechanical reinforcement, highlighting its promise for biomedical applications such as wound dressings and drug delivery systems.^48^ Cubogel-based ocular drug delivery systems have been extensively studied for enhancing drug bioavailability, permeability, and retention in the eye. Alhakamy et al. developed Cubogels^49^ with voriconazole permeability and antifungal activity, and they show promises in treating inflammatory and infectious diseases beyond ocular applications. In periodontitis treatment, Zheng et al. formulated azithromycin-loaded Cubogels with prolonged release and improved efficacy over commercial gels.^50^ Elgendy et al. repurposed atorvastatin in eugenol-enriched PEGylated Cubogels, showing significant clinical improvements in periodontal inflammation.^51^

In wound healing, soft gels provide a moist protective environment that promotes tissue regeneration while preventing infection. Functional gels loaded with antimicrobial agents, growth factors, or bioactive nanoparticles can further accelerate healing and reduce scarring. We developed a novel antibacterial and self-healable hydrogel film from biocompatible polymeric mixtures.^31^ The transparent hydrogel film was prepared by mixing polymeric blend solutions, such as carboxymethyl cellulose, sodium alginate, and PVA in aqueous media; it shows self-healing properties to restore its original functionalities and maintain intact structures after external damage (Figure 7a–d). The fabricated hydrogel film showed stable blood clots (within 119 ± 15 s) with rapid hemostasis (<2%) properties and effective antibacterial properties. The obtained hydrogel film also showed excellent cell viability on mouse fibroblast cells.

**Figure 7:**
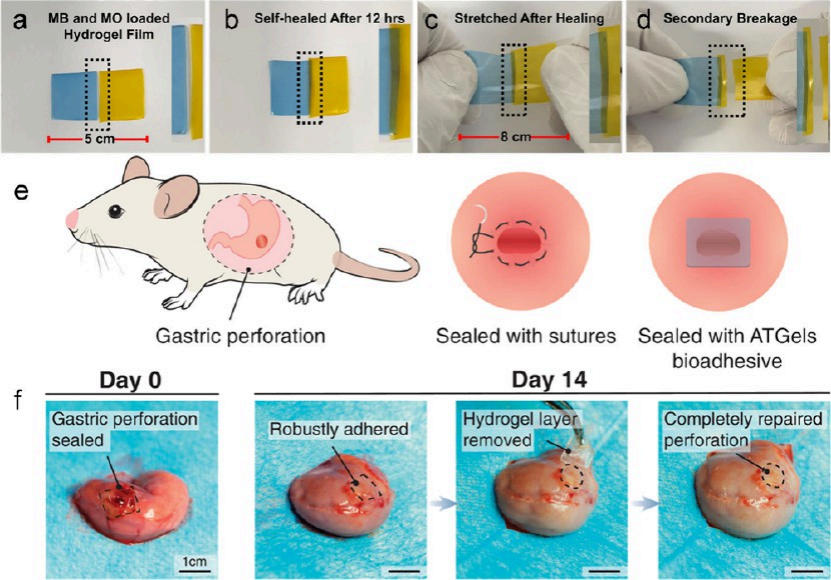
(a–d) Photographs of the self-healing abilities of the dried hydrogel film. (a) Methyl orange and methylene blue loaded two hydrogel films. (b) The hydrogel films were put together without external forces. (c) Both hydrogel films were completely healed after 12 h. (d) The film is stretched manually, and the breakage takes place on a different side, not on the healed side. Sutureless repair of gastric perforation in rat models. Reproduced from ref 31. Copyright 2023 American Chemical Society. (e) Schematic illustration for the gastric perforation repair with sutures or ATGel bioadhesive in rat stomachs. (f) Images showing the application of ATGel bioadhesive for sealing a gastric perforation on day 0 and the repaired stomach on day 14. The ATGel adhesive remains strongly adhered to the stomach tissue after 14 days, and complete closure of the perforation is confirmed upon careful removal of the hydrogel (scale bar: 1 cm). Reproduced with permission from ref 52. Copyright 2022 Wiley-VCH.

Functional soft gels also serve as tissue engineering scaffolds, supporting cell attachment, proliferation, and differentiation. Their tunable porosity, mechanical strength, and bioactivity make them suitable for regenerating skin, cartilage, bone, and even more complex tissues, such as nerve and cardiac muscle. We reported an acid-tolerant hydrogel (ATGel) bioadhesive, which integrates two distinct components, an acid-tolerant hydrogel–substrate and an adhesive polymer brush layer.^52^ The ATGel bioadhesive enables instant, tough, fluid-tight, and sutureless sealing of gastric perforations. By forming robust adhesive interfaces even in direct contact with gastric fluids, it promotes accelerated healing in a rat model by alleviating inflammation, suppressing fibrosis formation, and enhancing angiogenesis (Figure 7e,f).

## 5 Conclusion and Perspectives

From a chemical engineering perspective, advancements in cross-linking chemistry and polymer functionalization are crucial for tuning the structural integrity, degradability, and interaction of gels with biological systems. Dynamic and reversible cross-linking strategies (e.g., supramolecular, ionic, or enzymatic bonding) enable gels that heal themselves, reshape over time, or degrade in a controlled fashion. Covalent adaptable networks and click chemistry make it easier to tailor gels with precise architecture and functionality. Meanwhile, bio-orthogonal modifications allow for site-specific functionalization without disrupting biological processes. Such a process enables a one stop fabrication for the future medicine and/or drug delivery system, which could be supported with advanced machine learning and AI for the efficient material/system design.

AI and machine learning are increasingly valuable tools for the discovery and design of functional soft gels. These materials exhibit complex, multiscale structure–property relationships, which are difficult to capture using traditional trial-and-error approaches. AI/ML algorithms can analyze large experimental and computational data sets to identify patterns between gel composition, processing parameters, and mechanical performance, especially accurate quantitative responses like shape morphing to environment stimuli.^53–55^ Zheng et al.^56^ used PredNet to predict the hydrogel fracture behavior, and by training the ML model on simulation data generated from a multiscale hydrogel fracture model, the model was able to achieve a high level of accuracy in predicting fracture paths and crack propagation for various loading conditions.

On the fabrication front, the integration of gels with advanced manufacturing techniques such as 3D and 4D printing, electrospinning, microfluidic templating, and nanoimprint lithography is opening new avenues for customizing gel shape, structure, and functionality at the micro- and nanoscale. 3D and 4D printing provide powerful platforms for the discovery and development of functional soft gels by enabling precise spatial control and dynamic tuning of gel architectures. 3D printing allows the fabrication of soft gels with customized geometries, hierarchical porosity, and spatially patterned compositions. This structural control facilitates systematic exploration of how architecture influences properties such as mechanical strength, drug release, and cell–material interactions. 4D printing, by introducing time-dependent transformations (e.g., shape morphing, stimuli-responsiveness), expands this capability to functional gels that adapt to external cues such as temperature, pH, light, or biochemical signals. This enables the creation of dynamic, biomimetic systems that are closer to native tissue functions. These techniques allow researchers to design complex, patient-specific medical devices, including injectable depots, microneedles, and implantable scaffolds that respond to mechanical stress or physiological cues. Functional gels hold enormous potential as the foundation of the next generation of adaptive intelligent materials.

The research of functional gels is getting more and more exciting and transformative, driven by growing demands in healthcare, electronics, soft robotics, and sustainability. As researchers push the boundaries of gel innovation, we are witnessing a paradigm shift from passive materials toward adaptive, intelligent systems.
